# Vaccine wastage in The Gambia: a prospective observational study

**DOI:** 10.1186/s12889-018-5762-5

**Published:** 2018-07-11

**Authors:** Effua Usuf, Grant Mackenzie, Lamin Ceesay, Dawda Sowe, Beate Kampmann, Anna Roca

**Affiliations:** 1 Medical Research Council Unit The Gambia at London School of Hygiene and Tropical Medicine, Fajara, The Gambia; 2 Faculty of Infectious and Tropical Diseases, London School of Hygiene & Tropical Medicine, London, UK; 3 Murdoch Children’s Research Institute, Melbourne, Australia; 4 Expanded Programme on Immunization, Ministry of Health, Kotu, The Gambia; 5 Department of Paediatrics, St Mary’s Campus, Imperial College of Science, London, UK; 6 Faculty of Epidemiology and Population Health, London School of Hygiene & Tropical Medicine, London, UK

## Abstract

**Background:**

Vaccination is a cost-effective and life-saving intervention. Recently several new, but more expensive vaccines have become part of immunization programmes in low and middle income countries (LMIC). Monitoring vaccine wastage helps to improve vaccine forecasting and minimise wastage. As the costs of vaccination increases better vaccine management is essential. Many LMIC however do not consistently monitor vaccine wastage.

**Methods:**

We conducted two surveys in health facilities in rural and urban Gambia; 1) a prospective six months survey in two regions to estimate vaccine wastage rates and type of wastage for each of the vaccines administered by the Expanded programme on Immunization (EPI) and 2) a nationwide cross sectional survey of health workers from randomly selected facilities to assess knowledge, attitude and practice on vaccine waste management. We used WHO recommended forms and standard questionnaires. Wastage rates were compared to EPI targets.

**Results:**

Wastage rates for the lyophilised vaccines BCG, Measles and Yellow Fever ranged from 18.5–79.0%, 0–30.9% and 0–55.0% respectively, mainly through unused doses at the end of an immunization session.

Wastage from the liquid vaccines multi-dose/ single dose vials were minimal, with peaks due to expiry or breakage of the vaccine diluent.

We interviewed 80 health workers and observed good knowledge. Batching children for BCG was uncommon (19%) whereas most health workers (73.4%) will open a vial as needed.

**Conclusion:**

National projected wastage targets were met for the multi-dose/single dose vials, but for lyophilised vaccines, the target was only met in the largest major health facility.

## Background

Vaccination will save more than 20 million lives in low and middle income countries between 2001 2020 [1]. In recent years a number of new vaccines have been added to national vaccination programmes and consequently the cost per fully immunised child has increased considerably [2–4]. These growing costs make the considerate use of vaccines pressing and interest to minimise vaccine wastage has risen.

Vaccine wastage, usually measured as rate, is the proportion of vaccine doses supplied but not administered. Wastage is categorized primarily into two types: a) wastage of the remaining doses in opened vials at the end of an immunisation session, and b) wastage from unopened vials generally due to problems related to the cold chain, breakage or expiry [5, 6]. A number of factors are known to influence vaccine wastage. These broadly include the vaccines themselves, syringes, logistics, immunisation practices, and national policies [6].

In 2005, the WHO estimated that approximately half of the vaccines produced globally are wasted and therefore recommended that countries strengthen local vaccine wastage monitoring [6]. Parmar et al. reported in 2010 that only 19 (26%) of 72 GAVI eligible countries had submitted to WHO wastage data that could be analysed [7]. In the absence of local data, countries use WHO projected wastage rates to estimate their vaccine needs [8].

Due to restricted cold storage capacity in many developing countries, multi-dose vials are commonly used. In 2014, WHO revised its multi-dose vial policy (MDVP) to advice countries on minimising vaccine wastage while ensuring vaccine safety [9]. Under the policy, multi-dose vials with preservatives may be kept for up to 28 days after opening in contrast to the lyophilised vaccines, which do not contain preservatives, and should be discarded 6 h after reconstitution [9].

Despite the recommendations and renewed policy, recent data on vaccine wastage are lacking and published studies have relied primarily on mathematical models [10]. In this study, we prospectively quantified wastage rates and type for the vaccines used in the Gambian Expanded Programme on Immunization (EPI) and assessed the knowledge, attitude and practice (KAP) of Gambian health workers on general knowledge on immunisation and vaccine waste management.

## Methods

### Background setting

The Gambia a small country in West-Africa, had a population of about two million and a birth cohort of 86,990 live births in 2016. The national EPI was launched in 1979 initially with six vaccines and since then several other vaccines have been introduced (Table 1). Vaccination occurs mainly via fixed based and mobile outreach clinics, with occasional campaigns and national immunisation days as needed. Data collected from health and demographic surveillance systems between 2005 and 2012 showed coverage of Bacillus Calmette–Guérin (BCG) and Diphtheria-Pertussis-Tetanus (DPT3) to be > 95 and > 80% respectively across all regions in the country [11]. Full immunisation (child received BCG, three doses of oral polio vaccine (OPV), three doses of DTP and measles vaccines by one year of age) was only 52% in one region between 2000 and 2010 [12].

**Table 1:** Vaccines in The Gambian Immunization schedule, 2016

Formulation	Vaccine	Vial size (no. of doses)	Total doses/child	Schedule (Months)
Lyophilised	BCG	20	1	0
	Yellow fever	10	1	9
	Measles	10	2	9 & 18
Liquid (Multi-dose vial)	Hepatitis B	10	1	0
	OPV	10	6	0, 2, 3, 4, 9 & 18
	Pentavalent	10	3	2, 3 & 4
	IPV	10	1	4
	DPT	10	1	18
	TT	20	Up to 5	Pregnancy
Liquid (Single dose vial)	PCV 13	1	3	2, 3 & 4
	Rotavirus	1	3	2, 3 & 4

*MDV* Multi-dose vial, *BCG* Bacillus Calmette–Guérin, *OPV* Oral Polio vaccine, *DPT* Diphtheria - whole cell Pertussis-Tetanus, Pentavalent (2009)- [DPT-Hepatitis B (1990)- *H. influenzae* type b (1997)], IPV-Inactivated Polio vaccine (2016), PCV – pneumococcal conjugate vaccine (PCV7–2009, PCV13–2011)) TT-Tetanus toxoid given to pregnant women, *HPV* Human papilloma virus, given to school going children as part of a demonstration project was not included in this study

**Fig. 1:**
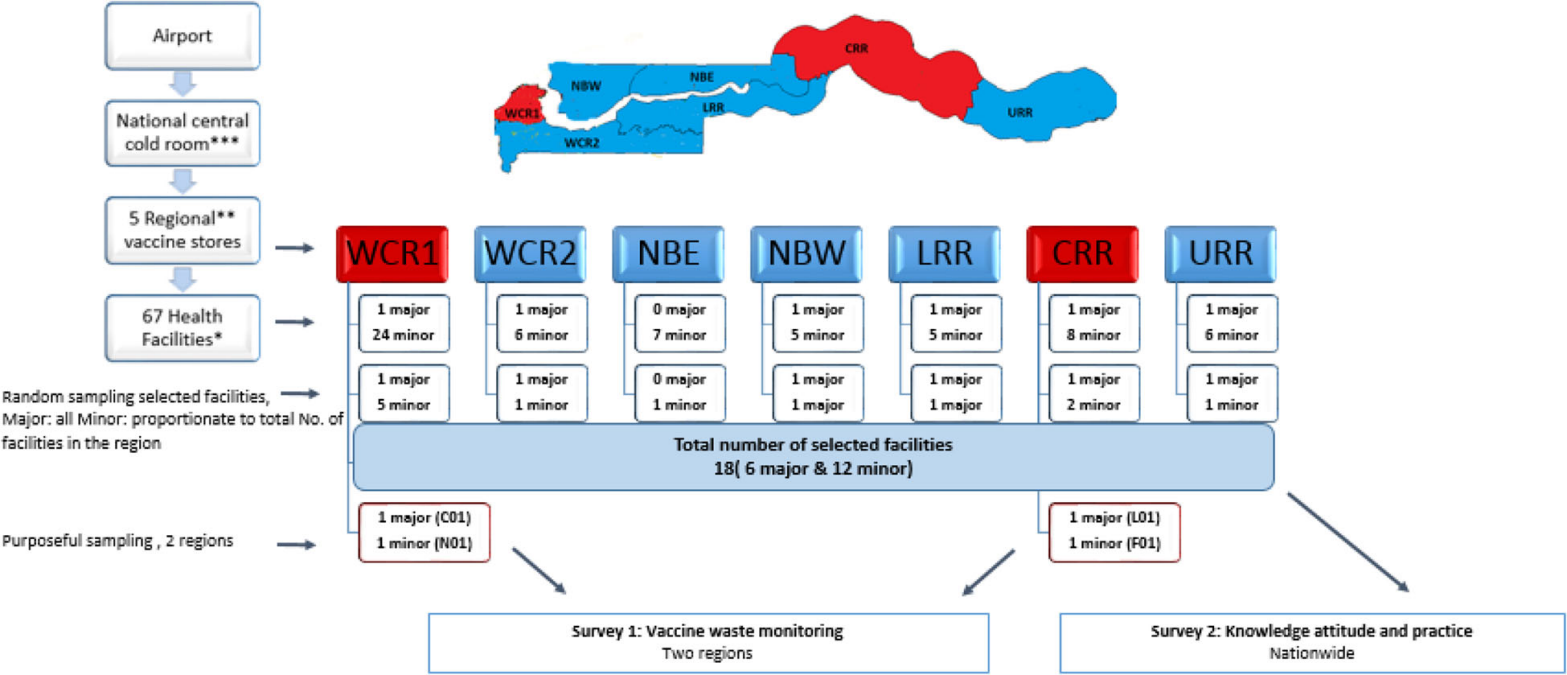
EPI collection & distribution system and study sampling framework. WCR West Coast Region, NBW North Bank West, NBE North Bank East, LRR Lower Region, CRR Central River Region, URR Upper River Region. *facilities with immunisation services, ** two without a store collect directly from national cold room, *** a second cold room in CRR

In The Gambia, vaccines are procured by UNICEF and are delivered by air twice a year, except for Rotavirus and Pneumococcal Conjugate Vaccine (PCV) which are delivered quarterly due to their large volumes. Once in the country, vaccines are stored at the central cold room and moved every quarter via a push system to the second cold room, in rural Gambia and five regional stores nationwide. Two of the regions without a store collect vaccines directly from the central cold room (Fig. 1).

In 2016 there were 67 health facilities providing vaccination services; 32 (47.8%) in urban and periurban areas. Fourteen out of the 67 facilities (20.9%) were private clinics. National EPI wastage projections were 35% for BCG, 5% for PCV13 and 15% for all other vaccines in the EPI schedule [13].

### Study design

We conducted two observational studies in a total of 18 selected health facilities (all six major health facilities with 110–150 beds per 150,000–200,000 population and 12 minor health facilities with 20–40 beds per 15,000 population) across rural and urban Gambia. The minor facilities were randomly selected proportionate to the total number of minor facilities in each region across the country (Fig. 1).

#### Survey 1 - Vaccine wastage in two regions

We purposefully selected two regions for this survey, West Coast Region (WCR1) and Central River Region (CRR) in urban and rural Gambia respectively (Fig. 1). These are the only regions with a cold room. In these regions, we selected the two major health facilities (one per region) and randomly selected two minor health facilities (also one per region) (Fig. 1).

Prospective data collection lasted for six months, from April to September 2016. WHO recommended forms were used for monitoring of vaccine wastage [6]. Trained study fieldworkers working closely with EPI personnel, recorded information at each health facility on the number of children immunised, doses discarded and the reason, and number of doses opened at each immunisation clinic covering both fixed and outreach sessions. At the beginning of each month, the fieldworkers with the EPI staff did a physical count of the total number of doses for each vaccine available at the facility. All data were collated at the end of the month.

Monthly data on doses received, doses issued and discarded were also captured from the two cold rooms in WCR1 and CRR.

#### Survey 2 – Knowledge attitude and practice among health workers nationwide

We conducted a cross sectional survey on the KAP of health care workers from 18 study facilities across all regions. All health workers engaged in vaccine delivery were eligible for participation. Health workers were interviewed on general knowledge on immunisation, and vaccine waste management using a pre-tested questionnaire. A separate interview was conducted with the senior officer in charge of EPI in each facility to collect data on general practices within the facility. The fieldworkers were trained not to read out the responses to questions but rather tick each that applied. At the end of the interview, the temperature chart and vaccine ledgers at the facility were inspected to observe the routinely captured data.

### Data analysis

We estimated vaccine wastage rate with 95% confidence interval for each vaccine included in the Gambia EPI schedule using the formula; Wastage rate = (Doses used – Children vaccinated)/ Doses used × 100 at the facility level. At the storage level, we measured the proportional wastage rate as number of doses discarded/ (start balance + number of doses received) × 100 [6].

Types of vaccine waste were described as a proportion for each vaccine i.e. number of each type of wastage recorded (e.g. number of doses discarded from broken vials) divided by the total number of doses discarded for that vaccine.

We described the practices at each facility as a percentage of the facilities that perform expected activities and described the KAP of the health workers. For each response we calculated the proportion that gave the correct response out of the total number of respondents.

## Results

### Characteristics of the facilities

All 18 study facilities had fixed base clinics at least once a month; 13 had between one and four sessions a month, four had more than one session a week and one had daily sessions. Outreach sessions occurred in all but two health facilities, one private and one minor health facility both in the urban area. The outreach sessions ranged from one to three times a week. All facilities requested vaccine supplies from their respective Regional Health Teams (RHT); 72.2% (13/18) on a monthly basis, ranging from weekly to quarterly.

### Vaccine wastage rates and type of wastage

#### Wastage from cold rooms

56,264 doses of Rotavirus vaccine expired from the central cold room (WCR1) and the regional cold room (CRR) in June 2016, with a proportional wastage rate of 29.4 and 6.7% respectively. There were also a total of 41,460 expired doses of Pentavalent vaccine from the two cold rooms. The highest proportional wastage rate was recorded for Yellow Fever (YF) vaccine in CRR due to breakage of the vaccine diluent, 62.7% (Table 2).

**Table 2:** Type of vaccine wastage at the cold rooms (April to September 2016)

Month	Vaccine	Type of wastage	Total doses discarded	Proportionate wastage (%)
*Urban*				
Apr	BCG	breakage	40	0.02
Apr	Measles	breakage	20	0.01
Apr	DPT	breakage	40	0.10
Apr	TT	breakage	20	0.01
Apr	Pentavalent	expiry	40,260	10.7
Jun	Rotavirus	expiry	55,428	29.4
*Rural*				
Apr	Pentavalent	expiry	1200	9.6
Jun	Rotavirus	expiry	836	6.7
Jun	BCG	missing	240	20.0
Jun	YF	missing	300	13.8
Aug	YF	Other^a^	1010	62.7

^a^breakage of vaccine diluent

#### Wastage in health facilities (four facilities)

**Fig. 2:**
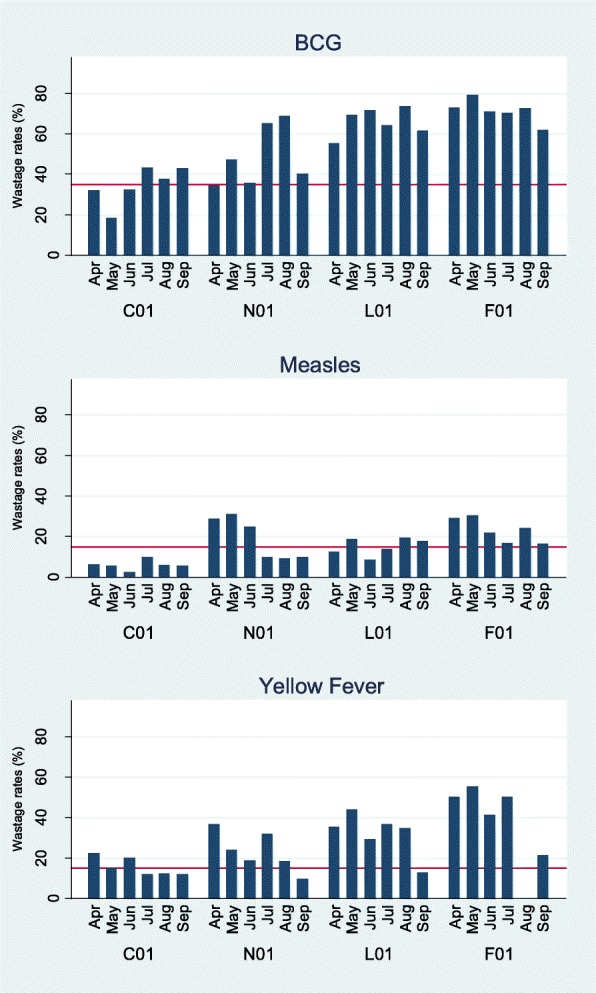
Wastage rates for Lyophilised vaccines. C01 Urban major, N01 urban minor, L01 Rural major, F01 Rural minor. Red line represents projected national wastage rate. YF was out of stock in F01 for August

We found a wide range of vaccine wastage rates depending on the vaccine or the facility. Vaccine wastage rates were highest for the lyophilised vaccines BCG, Measles and YF; mean (range) 54.9% (18.5–79.0%), 15.6% (0–30.9%) and 27.9% (0–55.0%), respectively (Fig. 2 and Table 3).

**Table 3:** Overall vaccine wastage in major and minor health facilities (April to September 2016)

Vaccine	Average wastage rate (%)			*P* value^a^
	Overall (95% CI)	Major	Minor	
Lyophilised				
BCG	54.9 (47.5–62.2)	50.0	59.7	0.631
Yellow fever	27.9 (21.9–33.9)	23.7	32.3	0.640
Measles	15.6 (11.9–19.3)	10.4	20.8	0.483
Liquid (MDV)				
Hep B	1.9 (0.2–3.5)	2.7	1.0	0.765
bOPV	4.4 (2.6–6.2)	6.2	2.6	0.663
Pentavalent	2.5 (−2.4–7.4)	0.13	4.8	0.460
IPV	5.1 (1.4–8.8)	3.2	7.0	0.672
DPT	0.1(−0.2–1.1)	0.9	0	0.747
TT	2.0 (−1.9–5.9)	0.3	3.7	0.548
Liquid (single)				
PCV13	0.1 (0.0–0.1)	0.1	0.02	0.945
Rotavirus	5.2 (0.1–10.3)	4.5	5.9	0.878

^a^Major versus minor, *MDV* multi-dose vial, tOPV switched to bOPV from mid-April,

**Fig. 3:**
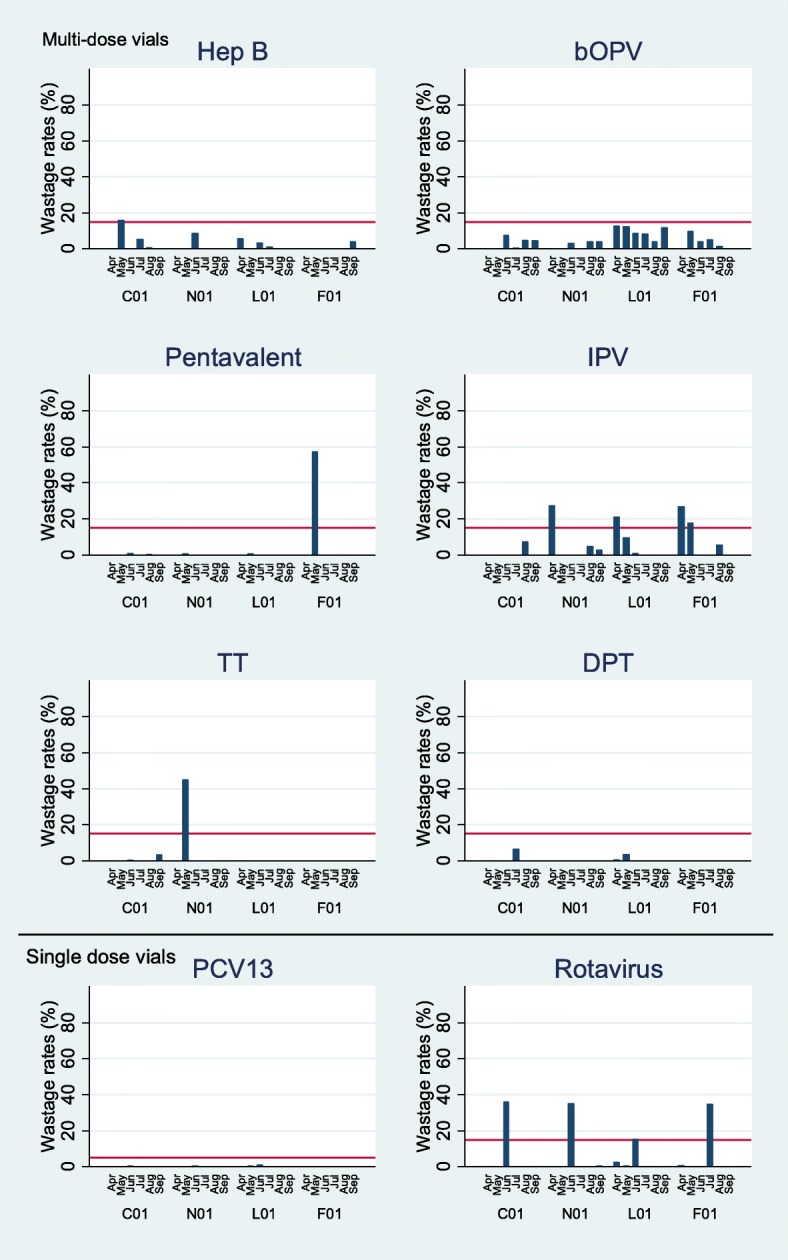
Wastage rates for Liquid vaccines. C01 Urban major, N01 urban minor, L01 Rural major, F01 Rural minor. Red line represents projected national wastage rate. PCV and Rota single dose vials MDVP does not apply

For the liquid vaccines for which the MDVP applies, bivalent OPV (bOPV) had the highest wastage ranging from 0 to 12.7%, with many doses unaccounted for (missing). Pentavalent wastage rate was < 1%, except for one facility that recorded 57.3% due to 160 expired doses in May 2016. For the other liquid vaccines, both multi/single dose vials, wastage rates were consistently low (Fig. 3a and b). PCV13 wastage rate was < 1% in all facilities during the six months survey.

Although there was a trend of higher rates of wastage for lyophilized vaccines in the major health facilities compared to the minor, this was not statistically significant (Table 3). There was no statistical difference between rural and urban facilities for any vaccine.

For all three lyophilised vaccines, more than 90% of the wasted doses for each vaccine was due to remaining doses from open vials that were discarded at the end of the immunisation session (Table 4). For liquid vaccines, the type of wastage varied. Rotavirus vaccines in stock expired in June/July 2016 at all facilities (Table 5). Other types of wastage recorded in unopened vials included missing inventory, vaccine vial monitor (VVM) failure, breakage and use of wrong diluent, however, these were minimal (Table 5). There was no record of wastage due to freezing during the entire study period.

**Table 4:** Type of wastage for Lyophilised vaccines

Vaccine	hf	Number of doses (Actual numbers)				Type of vaccine wastage (Percentages^a^)					
		Received	Opened	Immunised	Total wasted	Expiry	VVM	breakage	Missing	Unused	Other
Discard after 6 h											
BCG	C01	3660	3260	2172	1094	0	0	0	0.3	99.5	0.2
	F01	780	800	218	582	0	0	0	0	100	0
	L01	1300	1600	543	1057	0	0	0	0	99.7	0.3
	N01	820	860	469	411	0	0	4.9	0	95.1	0
Measles	C01	5400	5082	5020	230	0	0	0	0	100	0
	F01	750	720	579	173	0	0	0	0	94.2	5.8
	L01	1360	1660	1451	254	0	0	0	0	100	0
	N01	950	770	656	131	0	0	0	0	100	0
Yellow	C01	2560	2420	2098	376	0	0	2.7	0	94.7	2.6
fever	F01	490	500	291	223	0	0	4.5	0	95.5	0
	L01	970	990	650	339	0	0	0	0	97.9	2.1
	N01	500	520	431	120	0	0	0	0	91.7	8.3

tOPV switched to bOPV from mid-April, IPV introduced in Apr 2016 during the study, initially 10 dose without VVM, replaced with a 10-dose vial with VMM, others include spillage, breakage of vaccine diluent etc. hf health facility, ^a^percentage = (number doses wasted in each category/total wasted for that vaccine)X 100

**Table 5:** Type of wastage for Liquid vaccines

Vaccine	hf	Number of doses (Actual numbers)				Type of vaccine wastage (Percentages^a^)					
		Received	Opened	Immunised	Total wasted	Expiry	VVM	breakage	Missing	Unused	Other
MDVP											
Hep B	C01	2000	1935	2186	116	0	1.7	12.1	86.2	0	0
	F01	130	260	246	2	0	100	0	0	0	0
	L01	600	610	563	11	0	90.9	0	0	0	9.1
	N01	410	490	476	9	0	0	0	0	0	100
bOPV	C01	15,600	12,520	11,493	417	0	0	0	73.6	0	26.4
	F01	1860	1700	1466	58	0	0	0	25.9	0	74.1
	L01	4180	3940	3534	365	0	0	0	53.4	0	46.6
	N01	3740	2620	2378	57	0	35.1	0	0	0	64.9
Penta	C01	6210	6307	7049	12	0	0	83.3	0	0	16.7
	F01	890	680	738	160	100	0	0	0	0	0
	L01	2170	1920	1967	2	0	0	0	50	0	50
	N01	1500	1480	1600	1	0	0	0	0	0	100
IPV	C01	2430	2030	2199	34	0	0	0	88.2	0	11.8
	F01	270	280	263	35	5.7	0	0	0	94.3	0
	L01	770	710	683	46	0	2.2	0	0	97.8	0
	N01	500	550	543	28	0	0	0	0	71.4	28.6
DPT	C01	2180	2050	2310	20	0	0	0	100	0	0
	F01	270	240	250	0	0	0	0	0	0	0
	L01	910	830	826	5	0	0	0	100	0	0
	N01	300	270	238	0	0	0	0	0	0	0
TT	C01	3540	1890	1780	11	0	0	90.9	0	0	9.1
	F01	200	200	198	0	0	0	0	0	0	0
	L01	760	680	621	0	0	0	0	0	0	0
	N01	1500	420	457	80	0	0	0	100	0	0
MDVP NA^a^											
PCV13	C01	5199	5976	5975	1	0	0	0	0	0	100
	F01	840	796	796	0	0	0	0	0	0	0
	L01	2061	1968	1968	4	0	0	0	100	0	0
	N01	1550	1608	1607	1	0	0	0	0	0	100
	N01	3740	2620	2378	57	0	35.1	0	0	0	64.9
Rotavirus	C01	8520	7034	7041	762	99.7	0	0	0	0	0.3
	F01	894	776	775	70	98.6	0	0	0	0	1.4
	L01	2063	1933	1897	77	87.0	0	0	13.0	0	0
	N01	1751	1672	1666	202	97.5	0	0	2.5	0	0

tOPV switched to bOPV from mid-April, IPV introduced in Apr 2016 during the study, initially 10 dose without VVM, replaced with a 10-dose vial with VMM, others include spillage, breakage of vaccine diluent etc. hf health facility, ^a^percentage = (number doses wasted in each category/total wasted for that vaccine)X 100, MDVP multi-dose vial policy, NA not applicable

### KAP among health workers from 18 health facilities

Overall there were 82 health workers in the 18 selected health facilities. All were approached and 80 (97.6%) were interviewed. The other two travelled from their base facility and were unable to grant an interview after three attempts. Most health workers were male within the 20–29 years age group (73.8%, 59/80) and either public health officers [PHO] (67.5%, 54/80) or assistant PHO (25.0%, 25/80).

Almost all the health workers knew that measles vaccine had to be discarded after six hours of opening (93.6%, 73/78). Only 19.0% (15/79) said they would batch children for BCG vaccination and even less so for other vaccines. 73.4% (58/79) said they would open a vaccine according to the MDVP as soon as there was a request. All participants knew that aseptic conditions (79/79) were required and 94.9% (75/79) mentioned that an intact VVM must be in place before reusing a vaccine (Table 6).

**Table 6:** Knowledge attitude and practice among health workers

	Yes/total respondents (%)
Q. For how long can an opened measles vial be kept?	
2 h	1/78(1.3)
*6 h*	73/78(93.6)
24 h	3/78(3.8)
Don’t know	1/78(1.3)
Q. Do you batch/group children for any vaccine?	
BCG	15/79(19.0)
Hep B	5/79(6.3)
Measles	5/79(6.3)
YF	3/79(3.8)
OPV	3/79(3.8)
Q. For which vaccines does the MDVP apply?	
BCG	10/77(13.0)
*Hep B*^a^	51/80(63.8)
*OPV*^a^	52/80(65.0)
Rota	2/79(2.5)
*Penta*^a^	64/80(80.0)
*IPV*^a^	50/80(62.5)
PCV	3/80(3.75)
Measles	13/80(16.25)
Yellow fever	13/80(16.25)
*DTP*^a^	56/80(70.0)
*TT*^a^	55/80(68.75)
Q. What do you do to implement MDVP?	
Open as soon as requested	58/79(73.4)
Wait for a few children	9/79 (11.4)
Have a min number of children	2/79 (2.5)
Only on certain dates	3/79(3.8)
Others	37/78 (47.4)
Q. Conditions for reuse of vaccines	
Expiry date not passed	57/79 (72.2)
Appropriate cold chain conditions	34/79(43.0)
Aseptic technique	79/79(100.0)
VVM 1 or 2	75/79(94.9)
Others (Label intact)	37/78 (47.4)
Q. What reasons for wastage do you know?	
Expired	44/79(55.7)
High temp VVM	47/79(59.5)
Freezing	12/70(15.2)
Spillage	19/79(24.1)
Breakage	47/9(59.5)
> 6 h open	28/79(35.4)
Discard after opening	26/79(27.9)
All doses can’t be used	22/79(27.9)
Others	39/79(49.4)
Q. In what ways can vaccine wastage be reduced?	
Improve stock Management	50/79(63.3)
Organise sessions	29/79(36.7)
Batch	11/78(14.1)
EEFO	14/79(17.7)
Minimise misuse	21/79(26.6)
Implement MDVP	23/79(29.1)
Others	51/79(64.7)

^a^Multi-dose vial policy (MDVP) applies, VVM Vaccine vial monitor EEFO earliest expiry first out

The majority 72.5% (58/80) did not know what EEFO (‘Earliest expiry first out’) stands for and among those who said they knew, only 25.9% (*n* = 15) gave the right response. Over half 60.6% (47/78) reported that they knew the national wastage targets, however amongst these only one person gave the right estimate for all three vaccines BCG, Pentavalent and Measles as requested.

Sixty-one (77.2%) interviewees had received training on vaccine wastage/management with a wide range of sources of information. The most frequently reported sources were the EPI/RHT (73.8%, 59/80), others included School of Public Health, EPI training manuals, internet, WHO guidelines, mobile applications, and monthly staff meetings.

### Vaccine stock management at the health facility level (18 health facilities)

The availability of national policy guidelines was confirmed by the study staff at each facility. In addition 77.8% of the facilities (14/18) had other training materials. Vaccines were returned to the cold chain as per the MDVP. In the majority (14/18) of facilities the officer in charge reported that wastage was calculated on a monthly basis, and in the remaining four facilities, it was calculated either annually (one facility), quarterly (two facilities), or daily (one facility).

In the six months prior to the survey, cold chain failures did not occur and vaccine stock out was rare, but was reported for BCG, OPV and YF for two, three and four facilities respectively. All facilities except one had a regular supervisory visit coordinated by the RHT in the 3 months prior to the survey.

Data available from the vaccine stock ledgers showed that all facilities recorded the dates when vaccines were received, the names of the vaccines, doses received, balance in stock and VVM status. The variables least recorded were doses returned unused, which was recorded by only five facilities (27.8%), and vaccines damaged, recorded by only three (16.78%).

## Discussions

To our knowledge, this is the first prospective study to show vaccine wastage rates in a GAVI eligible country. Target wastage rates projected by the national EPI were met for the liquid vaccines in multi-dose/single vials. However, for the lyophilised vaccines, targets were only met at the major urban health facility with the largest catchment area of the four facilities (target population 150,000).

Wastage rates were highest for BCG. Overall, the rates were higher than the national target of 35% but within range of the WHO target of 50% [6, 13]. Vaccine wastage for lyophilised vaccines including BCG has been shown to correlate with the size of the vaccination session, as the number of children vaccinated decreases, the wastage increases because unused doses at the end of the session are discarded [14, 15]. Wastage in open vials has also been shown to be related to the vial size, with larger vials size leading to higher wastage [16]. The health workers in our study reported not to batch children for vaccination, but rather most would ‘open a vial for every child’ which is in keeping with the high national BCG coverage and the WHO guidelines [6, 11], but contrary to the Nigerian experience where vaccinators batch for a lyophilised vaccine [17]. Still, wastage rates in The Gambia were lower than in Bangladesh [14] with different size of the vaccination session and similar to India even though the facilities in India used 10-dose vials [15, 16, 18] compared to our 20-dose vial. A UNICEF review of the cost-effectiveness of changing from a 20-dose vial to a 10-dose vial BCG concluded that it may be more economical to waste than to reduce vial size because the given price for a 10-dose vial was only 2–8% lower than that of a 20-dose vial [19]. More recent models comparing 10 versus 5-dose vials for other vaccines have shown that a change to smaller vials could reduce wastage but this does not necessarily imply a reduction in costs as other technical reasons such as higher manufacturing and storage costs need to be taken into consideration [20, 21].

Our data supports existing evidence that wastage rates are low for vaccines that follow the MDVP. Wastage rates were lower in centres where the MDVP was practised in Cameroon [22] and Bangladesh [14] compared to those that do not. Similarly, in urban Indian health facilities, wastage of OPV reduced by 50% after introducing the MDVP [23]. The wastage rates reported for the liquid vaccines, Pentavalent, bOPV and Hepatitis B in our study were lower than that from Asian studies [14, 24].

Our study confirms mathematical models showing that wastage in single dose vials PCV are minimal [7]. PCV13 in 4-dose vial presentation is now available in addition to the single dose vial [25]. Earlier studies have shown that for immunisation programmes in developing countries, while per-dose price of antigens in multi-dose vials are lower than single-dose vaccines, even moderate wastage rates can quickly negate this price gain [7, 26]. As the MDVP is applicable to the 4-dose vial, wastage is expected to be low in countries where it is introduced.

Doses wasted in unopened vials are not acceptable and should be minimised with better planning [6]. The observed peaks in wastage rates for the Pentavalent, Tetanus toxoid and Rotavirus vaccines may be one off events during our study. Continuous monitoring is needed to quickly detect unwanted errors that could compromise the programme. It was reassuring that there was no wastage due to freezing and doses discarded due to changes in the VVM were few, perhaps reflecting the efficiency of the cold chain system with regular supervisory visits [2]. We noted that bOPV type of wastage were mainly ‘missing’ or ‘other’ for example spillage and spitting of the vaccine. This is not surprising as bOPV is oral and it may be difficult to quantify the actual number of doses that remain in a vial at the end of a vaccination session.

The KAP survey showed high general knowledge on vaccine waste management among PHO in The Gambia. We did not take into account the time from training which would have been useful particularly for those who did not know EEFO and the wastage rates projected targets. Regular refresher trainings will assist to improve their knowledge on ways to reduce wastage.

The main limitation of our study was that the wastage survey included only two regions of the country, the two with cold rooms. Though the results may not be generalizable to the other five regions, it is noteworthy that the EPI operates similarly in all regions with the RHT coordinating all vaccination activities. On the contrary, by obtaining data from both major and minor health facilities in rural and urban Gambia we showed a wide range of wastage rates across different settings. We conducted the wastage survey prior to the KAP to avoid changes in attitudes among health workers as this may have affected reporting and observations of vaccine wastage, the primary objective of the study.

## Conclusion

This operational research shows that, in general, national targets for vaccine wastage in The Gambia were consistently met for liquid vaccines in multi-dose/single vials during the six months of study period. For lyophilised vaccines such as BCG, high wastage rates were detected from unused doses at the end of immunisation sessions. Given the generally high coverage of vaccines in the Gambia, wastage in unopened vials may be considered acceptable. Extra effort should be made to ensure wastage data, which were incomplete at the facilities are captured routinely. A longer period of surveillance will detect if the wastage rates from expiration and breakages are a recurrent issue.

The results from our study should prompt new estimates for the global wastage rates. In the era of new and more expensive vaccines, data on vaccine wastage are crucial for local vaccine forecasting and to validate models. We propose studies in different settings to compare data and contribute to the review of global vaccine wastage estimates. Moving forward, studies to assess if the concern of wastage hinders maximum vaccine coverage would be helpful.

## Abbreviations

BCG: Bacillus Calmette–Guérin
bOPV: bivalent oral polio vaccine
CRR: Central River Region
DPT: Diphtheria pertussis tetanus
EEFO: Earliest expiry first out
EPI: Expanded Programme on Immunisation
Hib: Haemophilus influenzae type b
KAP: Knowledge attitude practice
LMIC: Low and middle income country
LSHTM: London School of Hygiene and Tropical Medicine
MDVP: Multi-dose vial policy
MRCG: Medical Research Council Unit The Gambia
OPV: Oral polio vaccine
PCV: Pneumococcal conjugate vaccine
RHT: Regional health team
tOPV: trivalent oral polio vaccine
VVM: Vaccine vial monitor
WCR: West Coast Region
WHO: World Health Organisation
